# Seroimmunotyping of African swine fever virus

**DOI:** 10.3389/fmicb.2023.1225587

**Published:** 2023-09-22

**Authors:** Alexey D. Sereda, Sanzhi Namsrayn, Vladimir M. Balyshev, Mikhail E. Vlasov, Irina P. Sindryakova, Galina Koltsova, Denis V. Kolbasov

**Affiliations:** Federal Research Center for Virology and Microbiology (FRCVIM), Vladimir Region, Volginsky, Russia

**Keywords:** African swine fever virus, seroimmunotypes, type-specific pig serum, hemadsorption inhibiting test, immunological test

## Abstract

The extreme genetic and immunobiological heterogeneity exhibited by the African swine fever virus (ASFV) has been a significant impediment in the development of an efficacious vaccine against this disease. Consequently, the lack of internationally accepted protocols for the laboratory evaluation of candidate vaccines has become a major concern within the scientific community. The formulation of such protocols necessitates the establishment of a consensus at the international level on methods for the determination of homologous and heterologous isolates/strains of ASFV. The present article provides a comprehensive description of biological techniques employed in the classification of ASFV by seroimmunotypes. These techniques involve a holistic evaluation of ASFV isolates/strains based on their antigenic properties as determined by the hemadsorption inhibiting test (HAdI) using type-specific sera and an immunological test (IT) conducted on pigs inoculated with attenuated strains. The article outlines the methods for setting up the HAdI test, an IT on pigs, and the processes involved in the acquisition of type-specific serums for the HAdI test. It is pertinent to note that the definitive classification of seroimmunotype can only be ascertained after conducting an IT on pigs. The findings from the HAdI test or the phylogenetic analysis of the EP402R gene should be considered preliminary in nature.

## 1. Introduction

African swine fever (ASF) is a contagious septic disease of domestic pigs and wild boars. The course of the disease can be hyperacute, acute, subacute, and chronic (Detray, 1957; Boinas et al., 2004; Blome et al., 2020). The causative agent of ASF is a DNA-containing virus of the *Asfivirus* genus, *Asfarviridae* family (Borca et al., 1998; Viruses IC on T, 2011).

African swine fever is currently having an unprecedented spread globally and one of the reasons for this spread is the lack of a safe and effective registered vaccine against the disease. It is known that pigs that have recovered after infection with the African swine fever virus (ASFV) can be protected from disease and/or death if subsequently infected with related virulent isolates/strains (Plowright, 1986). In addition, pigs inoculated with naturally attenuated or laboratory-selected strains of ASFV, or recombinant viruses, can also be protected from the disease or, if ill, from death after the infection by homologous virulent isolates/strains (Rock, 2017; Gallardo et al., 2019; López et al., 2020).

A widely used ASFV classification system is based on genotyping and determining the phylogenetic relationship of various isolates/strains. Currently, the studied isolates/strains of the ASFV are distributed into 24 genotypes (Bastos et al., 2003; Nix et al., 2006; Achenbach et al., 2017). Although ASFV genotyping is useful for some purposes, it does not fully correlate with the available data on cross-protection and may have limited value for predicting the effectiveness of vaccine cross-protection (Plowright, 1986; King et al., 2011; Rock, 2017).

In 1960, Malmquist and Hay were the first to document the occurrence of hemadsorption during the replication of ASFV in primary cultures of porcine bone marrow cells (PBMC) and primary peripheral blood leukocytes of swine (PBLS). In addition to the phenomenon of hemadsorption, they found that blood serum obtained from ASFV-infected pigs inhibits hemadsorption in ASFV-infected cultures of PBLS and PBMC cells, but does not affect the cytopathic effect and reproduction of the virus (Malmquist and Hay, 1960). Based on the results of studying the antigenic and protective properties of isolates/strains, a seroimmunotype classification of the ASFV was developed (Malmquist, 1963; Sereda and Balyshev, 2011; Sereda et al., 2020).

In 1968, Coggins reported the isolation of non-hemadsorbing subpopulations of the ASF virus (Coggins, 1968a). Further studies have shown that the loss of the ability to induce hemadsorption for the ASF virus during reproduction in cell culture is a common phenomenon (Pan and Hess, 1985; Jori and Bastos, 2009; Ravaomanana et al., 2010). A number of researchers have noted that non-hemadsorbing strains of the ASF virus isolated in nature or obtained under laboratory conditions have low virulence and the ability to form immune protection against subsequent infection of pigs with homologous virulent hemadsorbing isolates (Pan, 1992; Sánchez-Cordón et al., 2017).

The seroimmunotype classification is based on the results of a comprehensive assessment of ASFV isolates/strains by antigenic properties in the HAdI test with type-specific sera and an IT inoculated with attenuated strains (Sereda et al., 2020). The isolates/strains of the ASFV available in the collection of the FRCVM are divided into nine (I-IX) seroimmunotypes, and three other small groups: isolates whose serological affiliation does not correspond to the results of the immunological test (group X), isolates heterogeneous in seroimmunotype relation (group XI), and yet untyped isolates (group XII; Sereda et al., 2020). Each of the nine seroimmunotypes includes virulent hemadsorbing strains of the ASFV, including reference, and, as a rule, natural or laboratory-attenuated strains/isolates that have low or no virulence and are of hemadsorbing or non-hemadsorbing phenotype. In the classification described above, non-hemadsorbing isolates/strains are differentiated only by the results of an IT.

In our opinion, it is important to establish unified international definitions for homologous and heterologous isolates/strains. Evaluation of candidate vaccines should be based on seroimmunotype classification of isolates/strains established in hemadsorption delay reaction and immunological tests. This article describes methods that are used for ASFV seroimmunotyping.

The following terms will be used with the following meaning: serotype of ASFV is a group of isolates/strains of ASFV formed based on the results of the HAdI test; immunotype of ASFV is a group of isolates/strains of ASFV based on the results of IT; seroimmunotype of ASFV is a group of isolates/strains formed based on the results of grouping by HAdI test and IT.

## 2. Materials and methods

It is important to ensure that all plasticware used in the experiment is of tissue culture-grade quality. Additionally, all reagents and buffers should be properly sterilized either by autoclaving or filtration methods.

### 2.1. Preparing PBLS

#### 2.1.1. Materials and equipment

In the experiments, use female or male pigs that are 3 to 4 months old, e.g., 30–40 kg piglets of the Large White Pig breed (Pigs). Place the animals in a BSL 3Ag laboratory. Conduct a 6-day acclimatization period before starting the study. Keep and euthanize pigs according to AVMA guidelines for the care and use of laboratory animals (National Research Council (US), 2011).

##### 2.1.1.1. Hardware

Laminar flow cabinet, CO_2_ incubator, refrigerator (4°C), analytical balance, pH meter, inverted microscope, centrifuge (capable of spinning 50 mL conical tubes), and hemocytometer.

##### 2.1.1.2. Consumables

Vacuum blood collection tube, anti-coagulation (EDTA; Chengdu PUTH Medical Plastics Packaging Co., China), polypropylene conical tubes (50 mL), serological pipettes (10 mL), automatic pipettes with a volume of 20–200 μL, 100–1000 μL, aerosol-resistant filter tips, Millex-HV syringe filter unit, and 0.45 μm, PVDF (SLS, England).

##### 2.1.1.3. Chemical reagents

1. Complete blood leukocytes growing media (CBLGM): The Eagle’s minimal essential medium (EMEM) containing 10% of fetal bovine serum (FBS), penicillin (100–200 IU/mL), and streptomycin (100–200 mg/mL).
2. Phosphate-buffered saline pH 7.2, divalent cation-free (PBS): Nacl – 8.0 g, KH_2_PO_4_, − 0.2 g, Na_2_HPO_4_ ×12 H_2_O – 2.9 g, and KCl – 0.2 g. distilled water to 1,000 mL. Check the pH before use. Store at 4°C.
3. Ficoll–Hypaque 1,077 g/cm^3^ (GE Healthcare, United States).
4. Distilled water.
5. Red blood cell (RBC) lysis buffer: 155 mM ammonium chloride, 12 mM sodium hydrogen carbonate, and 0.1 mM ethylenediaminetetraacetic acid. Sterilization by filtration.
6. Trypan blue 0.4% (w/v).

#### 2.1.2. Method

African swine fever virus productively infects monocyte/macrophage cells in the domestic pig. Here, we describe the culture of primary peripheral blood leukocytes of swine.

- Collect the required volume of fresh defibrinated pig blood into tubes containing an anticoagulant.
- Add 15 mL of Ficoll–Hypaque solution at 20–22°C to polypropylene conical tubes (50 mL) and overlay it with diluted pig blood in sterile PBS at a 1:1 ratio.
- Centrifuge the tubes at 1000×g for 30 min at 20–22°C with the brake off.
- Carefully aspirate the interphase layer using a serological pipette, transfer it to a new 50 mL tube, and then fill the tube with PBS to the desired volume.
- Centrifuge at 400×g for 10 min at 20–22°C.
- Remove the supernatant by carefully decanting or aspirating it, and then discard it appropriately. Add 5 mL of RBC lysis buffer to each tube, ensuring complete resuspension of the pellet, and incubate the tubes at 20–22°C for 5 min. After the incubation, add 40 mL of PBS and centrifuge the tubes at 400×g for 10 min at 20–22°C.
- Wash cells with PBS twice more. Resuspend cells in CBLGM at a volume 2.0–2.5 times greater than the initial blood volume.
- Mix 100 μL of the resuspended cells with 800 μL of PBS and 100 μL of trypan blue solution. Stir the mixture thoroughly. Proceed to count the number of clear (non-stained) cells using a suitable counting method, such as a hemocytometer. Based on the cell count, calculate the cell concentration using the appropriate formula or software. Adjust the volume of CBLGM cells to a concentration of 3.0–4.0 million cells/mL.

### 2.2. Preparing washed RBC

#### 2.2.1. Materials and equipment

Pigs (see 2.1.1).

##### 2.2.1.1. Hardware

Laminar flow cabinet, refrigerator (4°C), bench-top centrifuge.

##### 2.2.1.2. Consumables

Vacuum blood collection tube, anti-coagulation (EDTA; Chengdu PUTH Medical Plastics Packaging Co., China), 5 mL centrifuge tube with lid, serological pipettes (5 mL), automatic pipettes with a volume of 100–1000 μL, and aerosol-resistant filter tips.

##### 2.2.1.3. Chemical reagents

1. Complete blood leukocytes growing media: The EMEM containing 10% of FBS, penicillin (100–200 IU/mL), and streptomycin (100–200 mg/mL).
2. Phosphate-buffered saline pH 7.2, divalent cation-free: Nacl – 8.0 g, KH_2_PO_4_, − 0.2 g, Na_2_HPO_4_ х12 H_2_O – 2.9 g, and KCl – 0.2 g. distilled water to 1000 mL. Check the pH before use. Store at 4°C.

#### 2.2.2. Method

- Centrifuge 1,0 mL of swine whole blood at 400×g for 5 min at 20–22°C.
- Remove plasma and buffy coat layer.
- Resuspend the red cells in 30 mL PBS and invert the tube to mix.
- Centrifuge the sample at 400×g for 5 min at 20–22°C and carefully remove and discard the supernatant. Repeat the washing step two more times using PBS, following the same centrifugation conditions.
- Resuspend RBCs in 1.0 mL of CBLGM, and then dilute the resuspended RBCs in a 1:100 ratio to prepare a 1% solution.

### 2.3. Оbtaining type-specific pig serum for the hemadsorption inhibiting test

#### 2.3.1. Materials and equipment

Pigs (see 2.1.1), PBLS (see 2.1), ASFV strains seroimmunotype III: reference Mozambique-78, and attenuated MK-200.

##### 2.3.1.1. Hardware

Laminar flow cabinet, refrigerator (4°C), freezer (− 40°C), water bath, and thermometer.

##### 2.3.1.2. Consumables

Vacuum blood collection tube, clot activator (Chengdu PUTH Medical Plastics Packaging Co., China), automatic pipettes with a volume of 20–200 μL, 100–1000 μL, aerosol-resistant filter tips, sterile 48-well Nunc cell culture plate with TC treatment, and 1.0 and 10.0 mL syringes.

##### 2.3.1.3. Chemical reagents

1. Complete blood leukocytes growing media: The EMEM containing 10% of FBS, penicillin (100–200 IU/mL), and streptomycin (100–200 mg/mL).
2. 40% solution of phosphonoacetic acid [HOOCCH_2_P(O)(OH)_2_] in sterile water (PAA).

#### 2.3.2. Methods

There are three main methods for obtaining sera that are active in the HAdI test from pigs: (i) survivors of ASF in subacute forms (Malmquist, 1963; Balyshev et al., 2015), (ii) pigs successively inoculated with attenuated and virulent reference strains of ASFV (Balyshev et al., 2015; Imatdinov et al., 2019), and (iii) survivors of acute or subacute forms of ASF as a result of treatment with a chemical – phosphonoacetic acid (PAA; Zubairov et al., 2017).

The infectious activities of ASFV strains were determined by titration in PBLS [four wells for each tenfold dilution; World organization for animal health (2019)]. The results were examined by the presence of hemadsorption phenomenon after 5–7 days. The virus titers were calculated according to the method described by Kerber in Ashmarin’s modification and expressed in 50% hemadsorbing units per mL (HAU_50_/mL; Ashmarin et al., 1975).

##### 2.3.2.1. Serum from subacute ASF survivors

- Infect pigs with a reference strain of ASFV of the selected seroimmunotype. Out of 10 pigs that are kept together, intramuscularly inoculate two pigs with a reference strain Mozambique-78 of ASFV of selected seroimmunotype III at a dose of 10^2.0^–10^3.0^ HAU_50_.
- The remaining eight animals would be infected by contact. When it is 14–21 days post the infection, examine the blood serum of survivors of the subacute form of ASF for the presence of active antibodies in the HAdI test (See 2.4.1).
- Obtain blood serum from surviving animals on day 42 after infection and test it in HAdI. Typically, their titers range from 1:40 to 1:10240.

##### 2.3.2.2. Serum from pigs successively inoculated with attenuated and virulent reference strains of African swine fever virus

Usually, administration of attenuated ASFV strains into pigs in doses from 10^2.0^ to 10^6.0^ HAU_50_ does not induce the formation of hemadsorption-inhibiting antibodies. In some cases, natural and laboratory-obtained attenuated hemadsorbing strains that cause a chronic form of ASF induce the formation of HAdI antibodies in pigs with titers no higher than 1:20–1:80. The process of obtaining serums active in the HAdI test by the second method requires taking into account that, on the one hand, after infection with a virulent strain, immunized animals must survive, and on the other hand, they must get sick with the manifestation of characteristic clinical signs of ASF (fever for several days, refusal of feed, and hemorrhages on the skin; Coggins, 1968a).

- Intramuscularly inoculate eight pigs with an attenuated strain MK-200 of ASFV at a dose of 10^6.0^–10^7.0^ HAU_50_.
- After 7–14 days, intramuscularly inoculate animals with a homologous virulent reference strain Mozambique-78 of the ASFV at a dose of 10^2.0^–10^3.0^ HAU_50_.
- In pigs that survived the subacute form of ASF, 14–21 days after the disappearance of clinical signs of the disease, examine blood serum for the presence of antibodies active in the HAdI test (See 2.4.1).

##### 2.3.2.3. Serum from pigs after application of chemicals drugs with therapeutic effects against ASF

The sodium salt of phosphonoacetic acid (phosphonate) inhibits the DNA polymerase of viruses by binding to the pyrophosphate site. Viral polymerase is significantly more sensitive to this drug than swine DNA polymerase. Phosphonoacetic acid, phosphonoacetic acid complex with 7-amino-1,3,5- triazaadamantane, and potassium pyridine salt of phosphonoacetic acid all prevented mortality of more than 80% of infected animals vs. 100% mortality of animals in the control group. The use of phosphonoacetic acid in combination with metisazone under microepizootic conditions prevented mortality of all piglets that had contact with the diseased ones, in comparison to 100% mortality in untreated animals. The possibility of obtaining type-specific sera was established, which removed the step of attenuation of virulent strains. The result allowed to shorten the serum preparation time by 3–12 times, which might be crucial for serotyping the virus since attenuation of individual strains can take up to 6–12 months or more (Zubairov et al., 2017).

- Administer intramuscular injection of the selected seroimmunotype III reference strain, e.g., Mozambique-78, to four pigs. The inoculation should be carried out at a dose ranging from 10^2.0^ to 10^5.0^ HAU_50_, with the specific virus dose adjusted based on the virulence of the ASFV strain.
- Monitor the clinical signs and record temperature every day.
- Once the body temperature of the infected pigs starts to rise above 40°C and clinical signs of the disease become evident (usually around 2–3 days after infection), begin administering intramuscular injections of a 40% PAA solution. The recommended dosage is 100–150 mg/kg of body weight. Administer the injections twice a day for the first 3 days, and then switch to once-a-day administration for the subsequent 10 days.
- On day 14, following the last recorded temperature peak, proceed to exsanguinate the animals and collect the blood samples. The collected sera should then be subjected to analysis using the HAdI assay.

In the HAdI assay, the titer of anti-ASFV serum can range from 1:40 to 1: 640. Depending on the virulence of the ASF virus strain, mortality rates in affected animals can range up to 50%.

In all cases, the selected type-specific pig serums are incubated for 30 min at a temperature of 56°C, aliquoted, and stored at −40°C. When selecting type-specific sera, it is assumed that their activity with homologous seroimmunotype reference strains of the ASFV in the HAdI test (see 2.4.1) should be from 1:40 or more (up to 1:10240), and with heterologous ones the inhibition of hemadsorption is not manifested.

### 2.4. Serotyping of African swine fever virus in the hemadsorption inhibiting test

Serotyping of the ASFV in the HAdI test makes it possible to obtain a preliminary result on the seroimmunotype classification of the studied isolates relatively quickly, which saves money, effort, and time, compared to the conduction of an immunological test on animals. The HAdI test uses reference strains of nine ASFV seroimmunotypes and corresponding type-specific reference pig sera. There are two main modifications of the HAdI test formulation. The first one was proposed by Malmquist (1963). The second one was proposed by Coggins (1968a) in modification Vigario (Vigário et al., 1970; Balyshev et al., 2015). In this article, we present both main modifications.

#### 2.4.1. Determination of working dilutions of type-specific pig sera (working serum dilution, WSD)

In the HAdI test, type-specific pig sera are used in working dilutions corresponding to their doubled titers. The titer of type-specific pig sera is taken as the highest dilution that causes inhibition of hemadsorption, multiplied by the dilution factor of the serum in the culture medium. The HAdI test in micropanel tablets is performed in the working volume, of which 0.90 is a cell suspension, 0.05 is a virus-containing material, and 0.05 is the serum of interest.

##### 2.4.1.1. Materials and equipment

PBLS (see 2.1), RBC (see 2.2), ASFV strain, Mozambique-78, swine anti-ASFV serums (IS1, IS2) to strain Mozambique-78, positive sera III serotype (PS), and normal porcine serum (NS) treated for 30 min at a temperature of 56°C.

###### 2.4.1.1.1. Hardware

Laminar flow cabinet, CO_2_ incubator, refrigerator (4°C), freezer (− 40°C), inverted microscope, and water bath.

###### 2.4.1.1.2. Consumables

Multichannel pipettes, automatic pipettes with a volume of 20–200 μL, 100–1000 μL, aerosol-resistant filter tips, and a sterile 48-well Nunc cell culture plate with TC treatment.

###### 2.4.1.1.3. Chemical reagents

1. Complete blood leukocytes growing media: The EMEM containing 10% of FBS, penicillin (100–200 IU/mL), and streptomycin (100–200 mg/mL).
2. Phosphate-buffered saline pH 7.2, divalent cation-free: Nacl – 8.0 g, KH_2_PO_4_, − 0.2 g, Na_2_HPO_4_ ×12 H_2_O – 2.9 g, and KCl – 0.2 g. distilled water to 1,000 mL. Check the pH before use. Store at 4°C.

##### 2.4.1.2. Method

Determination of working dilutions of investigated type-specific pig serums III serotypes from pigs (IS1 and IS2) in the HAdI test. The HAdI test is performed using 48-well plastic micropanels.

1. On day 0, prepare a suspension of PBLS with a concentration of 3.0–4.0 million cells/mL, and add 10 mL of the suspension to each well of the 48-well plates. Then incubate culture plates in a CO_2_ incubator at 37°C for 3 days with 5% CO_2_ and 90% relative humidity.
2. On day 3, take the following steps:
a. Calculate the dilution of viral stock required to achieve a concentration of 10^2.5^–10^3.0^ HAU_50_/25 μL for the HAdI test.
b. Dilute the virus in CBLGM.
c. Evaluate the status of adherent cells, specifically monocytes/macrophages, in the culture. Perform a wash by pipetting the medium up and down using an automatic pipette equipped with 1000 μL filter tips. This step aims to remove any non-adherent cells. Carefully discard the medium, and repeat the washing process two additional times to ensure thorough removal of non-adherent cells. Finally, add 450 μL of CBLGM to each well. Leukocyte culture should consist of single flattened, transparent cells uniformly distributed on the surface of the culture tablets with a density of at least 400–500 cells per field of view under low magnification (ocular × 10, lens × 10).
d. Infect cells with ASFV strain, Mozambique-78, 10^2.0^–10^3.0^ HAU_50_/25 μL/well. Leave the «cell culture controls» row not infected (Table 1).
e. Incubate plates overnight at 37°C in CO_2_ incubator.
3. On day 4, take the following steps:
a. Prepare a separate 48-well plate, with two-fold serum dilutions (1:2 to 1:256) of IS1, IS2, PS, and NS in CBLGM. Dispense 100 μL of CBLGM into 8 wells, occupying four rows (A-H) of the 48-well plate. In rows 1–4 (Table 1), add 100 μL of swine serum to each well, creating a 1:2 dilution. Ensure duplicates of each sample are included. Additionally, include suitable positive and negative controls in the plate.
b. Add 25 μL of the serum dilutions to the appropriate wells with infected cells.
c. Incubate plates at 37°C for 2 h in a CO_2_ incubator.
d. To each well, add 50 μL of 1% swine RBC.
e. Incubate plates overnight at 37°C in a CO_2_ incubator.

**Table 1:** Determination of working serum dilution (WSD).

Probes	Serum dilutions								WSD
	A	B	C	D	E	F	G	H	
	1:2	1:4	1:8	1:16	1:32	1:64	1:128	1:256	
	Titers								
	1:40	1:80	1:160	1:320	1:640	1:1280	1:2560	1:5120	
IS1	─*	─	─	─	─	**+**	**+**	+**	1:320
IS2	─	─	─	─	**+**	**+**	**+**	**+**	1:160
PS	─	─	─	─	─	**+**	**+**	**+**	1:320
NS	**+**	**+**	**+**	**+**	**+**	**+**	**+**	**+**	No
Virus controls	**+**	**+**	**+**	**+**	**+**	**+**	**+**	**+**	
Cell culture controls	─	─	─	─	─	─	─	─	

─^*^, absence of hemadsorbtion. +^**^, presence of hemadsorbtion.

On day 5, read hemadsorption using an inverted microscope. Note: the maximum dilution of each serum at which HAdI is complete (no rosetting cells) and write down the value. Monocytes/macrophages infected with ASFV with no anti-ASFV serum display a representative hemadsorption pattern. The result interpretation is represented in Table 1. Thus, the WSD of swine blood sera for IS1 and IS2 are 1:320 and 1:160, respectively.

#### 2.4.2. The hemadsorption inhibiting test

The method of setting the HAdI test used at FRCVM differs from the method of Malmquist (1963) in that before setting the reaction, the cultures of the PBLS or PBMC cells are washed with a nutrient media to reduce the number of red blood cells and remove loose cells. The sequence of addition of virus and serum does not play a fundamental role in this modification, since 2–3 days pass before the manifestation of hemadsorption (Vigário et al., 1970; Balyshev et al., 2015). The HAdI test is carried out with a micro method in cultured plastic 48-well micropanels. The HAdI test is performed with the following controls: cell cultures (cell culture to assess the quality of cell culture), type-specific serums (for the absence of non-specific hemadsorption), reference strains of I-IX seroimmunotypes, and test isolates of ASFV (for the presence of characteristic hemadsorption). The test uses type-specific sera, the WSD of which is determined in a preliminary experiment with reference strains of the ASFV.

The HAdI test for determining the serotype of ASFV isolates is carried out using the method described below, using 48-well plastic micropanels.

##### 2.4.2.1. Materials and equipment

PBLS (see 2.1). For serotyping purposes, the following reference strains of ASFV were utilized: I – Lisbon-57, II – Congo-49, III – Mozambique-78, IV – France-32, V – TSP-80, VI – TS-7, VII – Uganda, VIII – Stavropol 01/08, and IX – Davis. These viruses were propagated through 1–2 passages in cultures of PBLS cells, resulting in a viral titer ranging from 10^6.0^ to 10^7.5^ HAU_50_/mL. In this study, we investigated the serotype classification of the Katanga-78 and Kaluga-20 isolates. Type-specific pig anti-ASFV serum of serotypes I-IX, with the WSD in the HAdI test not lower than 1:40 (as a rule, 1:80–1:320).

###### 2.4.2.1.1. Hardware

Laminar flow cabinet, CO_2_ incubator, refrigerator (4°C), freezer (− 40°C), and inverted microscope.

###### 2.4.2.1.2. Consumables

Multichannel pipettes, automatic pipettes with a volume of 20–200 μL, 100–1000 μL, aerosol-resistant filter tips, and sterile 48-well Nunc cell culture plate with TC treatment.

###### 2.4.2.1.3. Chemical reagents

Complete blood leukocytes growing media: The EMEM containing 10% of FBS, penicillin (100–200 IU/mL), and streptomycin (100–200 mg/mL).

##### 2.4.2.2. Method

1. On day 0, prepare a suspension of PBLS with a concentration of 3.0–4.0 million cells/mL. The working volume, 1.0 mL, of the prepared suspension of cells is introduced into each well of culture plates, and into each well of the 48-well plate. Culture plates in a CO_2_ incubator with a CO_2_ content of 5% and a relative humidity of 90%, and incubate at a temperature of 37°C for 3 days.
2. On day 3, take the following steps:
a. Calculate the dilution of viral materials I-IX of seroimmunotypes to achieve a concentration of 10^2.0^–10^3.0^ HAU_50_/25 μL for the HAdI test (Table 2).
b. Dilute viruses in CBLGM.
c. Assess the status of adherent cells in each well of the plate. To eliminate non-adherent cells, perform a gentle wash by pipetting up and down in each well using an automatic pipette equipped with 1000 μL and discard the medium from the wells. Finally, add 450 μL of CBLGM to each well.
d. Infect cells with ASFV 10^2.5^–10^3.0^ HAU_50_/25 μL/well.
e. Calculate the dilution of reference sera so that after adding them to the wells in a volume of 25 μL, they reach the working dilutions.
f. Add 25 μL of the serum dilutions to the infected cells according to Table 2.
3. On days 4–7 read hemadsorption using an inverted microscope. Record the results. The results interpretation is represented in Table 2. Thus, the investigated isolate Katanga-78 belongs to the serotype I and isolate Kaluga-20 belongs to the serotype VIII. HAdI is taken into account after 48–72 h in the presence of well-expressed hemadsorption in the virus controls (at least 2–5 cells with specific hemadsorption in the field of view of the microscope, 400×) and its absence in the controls of type-specific sera and cell culture. Type-specific sera should inhibit the hemadsorption of homologous reference strains of ASFV and should not inhibit the hemadsorption of heterologous reference strains. Inhibition of hemadsorption of the studied isolate by one of the nine type-specific reference sera indicates that it belongs to the virus serotype for which this serum was obtained. The scheme of setting the HAdI test with the ASFV is shown in Table 2. Note,

**Table 2:** Serotyping scheme of the studied African swine fever virus (ASFV) isolates in the hemadsorption inhibiting test (HAdI).

Strains, isolates, and controls	Serotypes of type-specific pig serum									Virus controls	Serotype of strain/isolate
	1	2	3	4	5	6	7	8	9		
Lisbon-57	─	**+**	**+**	**+**	**+**	**+**	**+**	**+**	**+**	**+**	1
Congo-73	**+**	─	**+**	**+**	**+**	**+**	**+**	**+**	**+**	**+**	2
Mozambique-78	**+**	**+**	─	**+**	**+**	**+**	**+**	**+**	**+**	**+**	3
France-32	**+**	**+**	**+**	─	**+**	**+**	**+**	**+**	**+**	**+**	4
TSP-80	**+**	**+**	**+**	**+**	─	**+**	**+**	**+**	**+**	**+**	5
TS-7	**+**	**+**	**+**	**+**	**+**	─	**+**	**+**	**+**	**+**	6
Uganda	**+**	**+**	**+**	**+**	**+**	**+**	─	**+**	**+**	**+**	7
Stavropol 01/08	**+**	**+**	**+**	**+**	**+**	**+**	**+**	─	**+**	**+**	8
Davis	**+**	**+**	**+**	**+**	**+**	**+**	**+**	**+**	─	**+**	9
Katanga-78	─	**+**	**+**	**+**	**+**	**+**	**+**	**+**	**+**	**+**	1
Kaluga-20	**+**	**+**	**+**	**+**	**+**	**+**	**+**	─	**+**	**+**	8
Serum controls	─	─	─	─	─	─	─	─	─	─	─
Cell culture controls	─	─	─	─	─	─	─	─	─	─	─

(+), presence of 2–5 cells with specific HA in the view field of a microscope, 400×; (─), absence of cells with specific HA.

###### 2.4.2.2.1. Note

1. In the absence of inhibition of hemadsorption with reference sera, the test virus should be temporarily assigned to the group of untyped ASFV isolates.
2. When preparing for serotyping in HAdI, it is important to follow the recommendation to use the appropriate reference virulent strains of the ASFV to determine the activity of type-specific sera. The use of attenuated virus strains of the same seroimmunotype for this purpose may distort the result. It was found that the titers of type-specific sera in HAdI with virulent strains were 1.7–2.8 log_2_ lower than with the attenuated strains. The highest differences, by 29.5 times, were noted with the attenuated strain FK-32/135 inducing “loose” hemadsorption and with the virulent strain France-32, belonging to the IV seroimmunotype, inducing “dense” hemadsorption (Sereda et al., 2016). These differences could be a consequence of differences between virulent and attenuated strains in the proportion of the circumference of erythrocytes in contact with the plasmalemma of infected macrophages during hemadsorption, or differences in the structure of strain populations based on the number of red blood cells attached to infected macrophages (Sereda et al., 2016).
3. For long-term storage, serums specific to the reference type are lyophilized.
4. To reduce the number of reference strains and sera used in animal seroimmunotyping, geographical isolation sites of the ASFV are preliminarily evaluated. For example, on the Iberian Peninsula, the presence of ASFV I and IV seroimmunotypes is possible, whereas in West Africa, the presence of the following seroimmunotypes is registered: ASFV I, II, and IV (Malogolovkin et al., 2015).

### 2.5. Immunological test

The seroimmunotype appurtenance of the studied isolates, previously assigned based on the results of the HAdI test to the same group with ASFV strains of the corresponding seroimmunotype, is determined by an immunological test on pigs (Balyshev et al., 2011).

#### 2.5.1. Materials and equipment

Pigs (see 2.1.1), PBLS (see 2.1).

##### 2.5.1.1. Hardware

Laminar flow cabinet, CO_2_ incubator, refrigerator (4°C), freezer (− 40°C), analytical balance, inverted microscope, and thermometer.

##### 2.5.1.2. Consumables

Multichannel pipettes, automatic pipettes with a volume of 20–200 μL, 100–1000 μL, aerosol-resistant filter tips, a sterile 48-well Nunc cell culture plate with TC treatment, and 1.0 mL syringes.

##### 2.5.1.3. Chemical reagents

Complete blood leukocytes growing media: The EMEM containing 10% of FBS, penicillin (100–200 IU/mL), and streptomycin (100–200 mg/mL).

#### 2.5.2. Method

- The infectious activity of the tested isolate, as well as the attenuated and virulent reference strains of the ASFV, is determined in the cultures of PBLS (See 2.3.2).
- Pigs (eight heads) are inoculated twice, intramuscularly, with an interval of 14 days with attenuated ASFV strain MK-200 at a dose of 10^6.0^–10^7.5^ HAU_50_.
- Then, after 28 days from the second inoculation, four pigs are intramuscularly infected with a virulent reference strain Mozambique-78 at a dose of 10^3.0^ HAU_50_, and four with the studied isolate at the same dose. To control virulence, an additional two intact pigs each are infected in a similar way with a virulent reference strain of the ASFV and a test isolate at a dose of 10^3.0^ HAU_50_.
- During the experiment all pigs are monitored in terms of body temperature and other clinical signs of ASF. As a rule, during this time, the death of control animals is observed. The specificity of the disease and death from ASF of experimental pigs is confirmed by the isolation of the virus in the culture of PBLS.
- In the absence of death of at least 3/4 of pigs inoculated with an attenuated strain and subsequently infected with a virulent reference strain or a test isolate of the ASFV, they are considered to have an immunological correspondence and belong to the same seroimmunotype.

##### 2.5.2.1. Note

1. In case the pigs inoculated with an attenuated strain and infected with a test isolate die from ASF, then this isolate does not have an immunological correspondence with the attenuated and virulent reference strains taken in the experiment. According to the existing classification, it should be assigned to the group of strains that do not match the results of serotyping in the HAdI test and in the immunological sample.
2. If intact pigs infected with the tested isolate of the virus (virulence control) did not show signs of disease, characteristic symptoms, and did not die from ASF, this indicates its low virulence. After 21 days, these pigs are infected with a virulent reference strain of ASF virus of the presumed serotype. In case of survival of the pigs, the tested isolate of ASF virus is classified as belonging to the same serotype as the reference strain used.

## 3. Discussion

### 3.1. Comparative analysis of the genotyping and seroimmunotyping of the African swine fever virus

In 2015, a new approach was proposed that makes it possible to predict with a high degree of probability the seroimmunotype belonging to both hemadsorbing and non-hemadsorbing isolates of the ASFV (Malogolovkin et al., 2015). It is based on sequencing and phylogenetic analysis of the EP402R gene coding the major glycoprotein CD2v of the ASFV, responsible for the phenomenon of hemadsorption during reproduction of the ASFV (Borca et al., 1998; Malogolovkin and Kolbasov, 2019). Now, the method of “serotyping” uses a short fragment of the EP402R gene 90 nucleotides long (Thanh et al., 2021). However, in our opinion, the results of “serotyping” based on the nt sequence of the EP402R gene should be necessarily confirmed by studies of the antigenic properties of isolates in the HAdI test and IT.

### 3.2. Determination of the immunotype of non-hemadsorbing African swine fever virus isolates

The HAdI test is not applicable to determine the type of non-hemadsorbing ASFV isolates. Therefore, only an IT is used. If the non-hemadsorbing isolate is virulent, then its type affiliation is determined in an immunological test on pigs previously inoculated with attenuated strains of the ASFV. If the studied non-hemadsorbing isolates of the ASFV are avirulent or weakly virulent, then they are used on vaccinated pigs twice with an interval of 14 days; then, after 28 days pigs are infected with virulent hemadsorbing reference strains of the ASFV of various seroimmunotypes.

### 3.3. Heterologous seroimmunotype isolates

The seroimmunotype classification has demonstrated its adequacy in studies on obtaining candidate live vaccines based on the selection of attenuated ASFV strains (Sereda et al., 2020). Another result of the application of the seroimmunotype classification was the proof of the heterogeneity of the Kiravira-67 isolate; it is a parent isolate to the four strains that belong to different seroimmunotypes: I, III, V, and VI (Sereda et al., 2014).

It should be noted that the final result of seroimmunotyping is achieved only as a result of setting an immunological test on pigs. Hemadsorption inhibiting test or phylogenetic analysis data on the EP402R gene should be considered preliminary. It was indicated above that the X group according to the seroimmunotype classification includes ASFV strains in which the HAdI results do not coincide with the IT (Sereda et al., 2020). A possible reason for this may be the formation of a mixed ASFV population consisting of isolates/strains of two or more seroimmunotypes. It has been experimentally established that when pigs are infected with mixtures of ASFV strains of two different seroimmunotypes, there is a persistent dominance in the manifestation of hemadsorption of one strain over the other (Sereda et al., 2014).

### 3.4. Exotic serotyping tests

By utilizing the radioimmunoprecipitation assay, researchers identified a significant virus-specific glycoprotein known as gp110-140 (also referred to as CD2v). This glycoprotein exhibited a molecular weight ranging from 110 to 140 kDa and displayed a characteristic dumbbell-shaped band, which is typical for highly glycosylated proteins (Sereda et al., 1993, 2018). For its detection, two principal conditions were required: (1) use of the metabolically ^3^H-glycosamine-labeled proteins derived from lysates of PBM naturally susceptible A-cells infected with ASFV hemadsorbing strains as an antigen source; and (2) use of the homologous antisera with high activity in HAdI as an antibody source. Through extensive investigations, the serotype specificity of gp110-140 has been successfully determined (Sereda et al., 2018). While using some homologous components in the radioimmunoprecipitation assay, the dumbbell-like bands of gp 110–140 manifested as the major ones. In the assays using heterologous components, gp 110–140 was not detected in the fluorograms or manifested less intensively as compared to the results of the homologous assay.

A method exists for quantitative assessment of the serological relationship of hemadsorbing ASFV strains. Results of radioimmunoprecipitation are to be recorded not through a visual examination of the fluorogram, but by the number of pulses per minute using a β-counter. ^3^H-glucosamine labeled gp 110–140 preparations, derived from hemadsorbing ASFV reference strains purified with ion-exchange chromatography on DEAE-Sephacel, were used as antigens for the quantitative version of the radioimmunoprecipitation procedure. The percentage of specific binding obtained with the control sera of intact pigs was not greater than 3%. The serological relationship of gp110-140 with serotype-heterologous antisera varied from 20 to 45% which indicates that the gp110-140 contains both homologous and heterologous epitopes (Sereda et al., 1998, 2018). Thus, the serotype specificity of gp110-140 was confirmed using two versions of radioimmunoprecipitation assay.

In summary, the described seroimmunotyping technique is based on the combination of two immunological tests *in vitro* and *in vivo*. It provides adequate experimental results during the development and evaluation of the protective properties of candidate vaccines against ASF. Knowledge about the seroimmunotype assignment of isolates/strains is useful in monitoring ASF, determining the phylogenetic relationships of virus isolates, and confirming the possible source of virus introduction.

## Data availability statement

The original contributions presented in the study are included in the article/supplementary material, further inquiries can be directed to the corresponding authors.

## Ethics statement

The manuscript presents research on animals that do not require ethical approval for their study.

## Author contributions

AS: writing—original draft preparation. DK: conceptualization, supervision, and project administration. SN and VB: writing—review and editing. MV, IS, and GK contributed to both the conception and design of the work. All authors contributed to the article and approved the submitted version.

## Conflict of interest

The authors declare that the research was conducted in the absence of any commercial or financial relationships that could be construed as a potential conflict of interest.

## Publisher’s note

All claims expressed in this article are solely those of the authors and do not necessarily represent those of their affiliated organizations, or those of the publisher, the editors and the reviewers. Any product that may be evaluated in this article, or claim that may be made by its manufacturer, is not guaranteed or endorsed by the publisher.
